# Supplementary material for: Chemical Composition of Volatile and Extractive Organic Compounds in the Inflorescence Litter of Five Species of Woody Plants
Source: Plants (Basel). 2024 Jul 3;13(13):1829. doi: 10.3390/plants13131829 (PMC11244211; doi:10.3390/plants13131829)
Supplement: Supplementary file 1 [file plants-13-01829-s001.zip › plants-3043830-supplementary.pdf]

Supplement of

Chemical Composition of Volatile and Extractive Organic Compounds in the Inflorescence Litter of Five Species of Woody Plants

Valery A. Isidorov * and Jolanta Masłowiecka

Institute of Forest Sciences, Białystok University of Technology, 15-351 Białystok, Poland; j.maslowiecka@pb.edu.pl
* Correspondence: isidorov@uwb.edu.pl

Table S1. Chemical composition (% of TIC) of VOCs emitted by fallen inflorescences of some forest-forming deciduous trees of the boreal and mid-latitude zones of Europe. *A* - freshly fallen inflorescences, *B* - after two weeks of decomposition in litter bags

Compound	CAS	RI^{Cal}	RI^{Db}	*Carpinus betulus*		*Populus tremula*		*Acer platanoides*		*Betula pendula*	
				A	*B*	*A*	*B*	*A*	*B*	*A*	*B*
Methanol	67-56-1	-	381	-*	-	3.21	-	-	-	-	-
Ethanol	64-17-5	-	484	-	-	-	-	-	-	1.15	trace**
Acetone	67-64-1	-	501	-	1.37	0.93	-	-	-	3.93	0.52
Diethyl ether	60-29-7	-	509	0.28	-		0.60	1.20	-	-	-
Formic acid	64-18-6	-	520	-	0.33	-	1.54	-	-	-	-
Isobutanal	78-84-2	-	555	-	0.86	-	-	-	-	-	-
α-Methylacroleine	78-85-3	-	568	-	trace	-	-	-	-	-	-
Vinyl acetate	108-05-4	-	580	-	0.64	-	-	-	-	-	-
n-Hexane	110-54-3	600	600	0.59	trace	6.50	trace	13.64	0.23	0.81	-
2-Methylfuran	534-22-5	606	610	0.06	-	-	-	-	trace	1.56	0.45
3-Methylfuran	930-27-8	612	613	0.59	-	2.11	-	2.37	0.10	trace	0.75
Isobutanol	78-83-1	622	617	-	-	1.06	-	-	-	-	-
Isobutyl nitrile	78-82-0	623	623	-	-	-	-	1.91	-	-	-
(E,Z)-2,4-Hexadiene	5194-50-3	635	635	-	-	-	-	-	-	trace	trace
Isopentanal	590-86-3	648	650	-	-	1.09	-	-	-	-	-
2-Methylbutanal	96-17-3	658	658	-	0.90	1.62	-		-	-	-
Acetic acid	64-19-7	661	665	0.37	1.89	4.00	1.71	2.37	158	-	2.47
1-Butanol	71-36-3	663	662	-	-	-	-	1.80	-	-	-
(*E*)-2-Butenal	123-73-9	664	665	-	1.27	-	-		-	-	-
5-Ethyl-2-hexene?***	3404-62-4	680	-	-	-	-	-	0.56	-	-	-
1-Penten-3-ol	616-25-1	680	682	-	0.26	0.83	-	-	-	-	-
1-Penten-3-one	1629-58-9	684	684	-	0.32	-	-	-	-	-	-
1,4-Heptadiene?	5675-22-9	696	-	-	-	-	0.37	-	-	-	-
n-Heptane	142-82-5	700	700	0.44	-	-	-	-	-	0.39	0.90
3-Pentanone	96-22-0	701	700	-	-	2.21	-	0.73	-	-	-
Pentanal	110-62-3	703	706	-	0.58	-	-		-	-	-
2-Ethylfuran	3208-16-0	705	704	-	0.53	trace	trace		-	trace	-
2-Methylbutanenitrile	18936-17-9	721	718	-	-	0.31	-	3.09	-	-	-
Isopentanol	123-51-3	725	726	-	0.15	-	-	-	0.66	-	-
3-Methylbutanenitrile	625-28-5	729	729	-	-	2.44	-	1.70	-	-	-
2-Methyl-1-butanol	137-32-6	732	729	-	-	6.06	-	-	-	-	-

(*E*)-3-Penten-2-one	3102-33-8			-	0.36	-	-	-	-	-	-
Dimethyldisulfide	624-92-0		739	-	0.25	1.01	-	-	-	-	-
Oxalic acid?	144-62-7	739	746	-	-	-	-	-	1.39	-	0.17
Pyridine	110-86-1	749	748	-	0.07	-	trace	-	-	-	-
(*E*)-2-Pentenal	1576-87-0	751	753	-	0.38	-	-	-	-	-	-
Toluene	624-92-0	762	762	16.28	0.63	5.63	6.47	3.26	11.80	-	5.57
NN (43,41,29,57,85)	-	768		-	-	-	-	-	-	0.50	-
(*Z*)-2-Penten-1-ol?	1576-95-0			-	0.16	-	-	-	-	-	-
3-Methyl-2-butenal?	107-86-8	771	772	-	0.09	-	-	-	-	-	
Methyl isopentanoate	556-24-1	775	772	-	-	0.49	-	-	-	-	
1-Octene	111-66-0	795	794	0.88	0.14	0.42	-	trace	0.48	-	trace
n-Octane	111-65-9	800	800	1.03	-	2.08	-	-	0.43	-	0.21
Hexanal	66-25-1	802	802		2.93	trace	-	-	-	-	0.59
3-Hexenal	4440-65-7	806	803	-	-	-	-	0.69	-	-	-
Alkadiene C_8H_{14} (81,86,67, 55,110)	-	812	-	-	-	-	-	-	0.03	-	-
Isovaleric acid	503-74-2	817	838	-	-	-	-	-	-	0.46	-
1,3-Octadiene	1002-33-1	822	823	1.03	-	-	0.38	-	-	-	0.37
NN	-	824	-	0.85	-	-	-	-	-	-	-
2-Methylbutyric acid	116-53-0	832	839	-	-	-	-	-	-	0.64	-
Furfural	98-01-1	833	833		0.09	-	-	-	-	-	-
C9H14 (107,91,122,105,79)	-	840	-	-	-	-	-	-	0.14	-	-
NN (109,124,43,79,110)	-	849	-	-	-	-	-	-	-	-	0.56
(2*E*)-Hexanal	6728-26-3	852	855	-	2.62	-	-	0.26	-	-	-
(*Z*)-3-Hexen-1-ol	928-96-1	854	857	-	-	0.35	-	0.71	-	-	-
NN (109,124,43,79,110)	-	856	-	-	-	-	-	-	0.37	-	-
Ethyl benzene	100-41-4	858	859	trace	-	-	-	-	-	-	trace
(*E*)-2-Hexen-1-ol?	928-95-0	865	865	-	0.10	-	-	0.41	-	-	-
p-Xylene	106-42-3	866	865	0.29	-	-	0.74	-	0.14	-	trace
1-Hexanol	111-27-3	867	866	0.25	0.45	0.73	-	0.61	-	-	-
1,3,5-Octatriene	40087-61-4	877	880	trace	-	-	-	-	-	-	0.71
NN (81,80,124,79,82)	-	889	-	-	-	-	-	-	-	-	0.41
n-Butylfuran	4466-24-4	880	885	trace	-	-	-		-	-	-
NN (81,43,57,109,79)	-	880	-		0.10	-	-	-	0.14	-	-
NN (119,91,79,77,120)	-	886	-	-	-	-	0.68	-	-	-	-
Styrene	100-42-5	890	890	????	1.04	15.36	24.81	-	1.27	-	-
2-Heptanone	110-43-0	890	891	-	-	-	-	1.83	-	-	-
m-Xylene	108-38-3	892	891	0.43	-	-	-	-	0.40	-	-
1-Nonene	124-11-8	896	896	0.45	trace		-	-	trace	-	-
n-Nonane	111-84-2	900	900	1.06	0.31	2.21	-	trace	trace	-	-
Heptanal	111-71-7	902	902	trace	0.63	-	-	-	-	-	-
NN (43,41,55,71,29)	-	904	-	-	-	0.61	-	-	-	-	-
2-Heptanol	543-49-7	906	904	-	-	-	-	0.33	-	-	-
(*E,E*)-2.4-Hexadienal	142-83-6	910	911	-	0.52	-	-	-	-	-	-
Anisole	100-66-3	916	918	-	-	-	0.39	-	0.14	-	-
Tricyclene	508-32-7	919	920	-	-	0.23	0.58	trace	-	-	-
3-Thujene	2867-05-2	925	926	-	-	trace	0.30	2.17	-	-	0.39
α-Pinene	80-56-8	930	932	1.10	0.06	6.81	0.51	2.82	0.19	4.42	1.31
NN (69,41,68,109,67)	-	936	-	-	-	0.26	-	-	-	-	-
NN (**122**,107,121,79,77)	-	934	-	-	0.16	-	-	-	-	-	0.85

Hydrocarbon C_9H_{16}?	-	943	-	-	0.15	-	-	-	-	-	0.30
NN (69,41,68,109,67)	-	944	-	-	-	0.83	0.13	-	-	-	-
Camphene	79-92-5	944	946	-	-	0.72	0.52	trace	-	trace	0.24
NN (83,69,84,55,41)	-	945	-	-	0.10	-	-	-	-	-	-
Verbenene	4080-46-0	951	952	-	-	-	trace	-	-	2.04	-
C10H20 (56,69,57,55..140)	-	954	-	-	-	-	-	-	0.44	-	-
(*Z*)-2-Heptenal	57266-86-1	955	954	-	2.69	-	-	-	-	-	-
NN 93,111,55,67,43_	-	956	-	-	-	-	-	-	0.24	-	-
Benzaldehyde	100-52-7	959	961	-	1.67	0.68	-	-	-	-	-
Dinethyltrisulfide	3658-80-8	966	973	-	0.20	0.31	-	-	-	-	-
3,7,7-Trimethyl-1,3,5-cyclopentatriene	3479-89-8	967	970	-	-	-	2.35	-	-	-	-
NN (57,55,71,97,41)	-	969	-	-	0.14	-	-	-	-	-	-
Sabinene	3387-41-4	971	973	-	0.29	0.36	trace	10.97	trace	-	trace
β-Pinene	127-91-3	973	975	trace	0.28	0.50	0.29	0.72	trace	2.58	0.80
1-Octen-3-one	4312-99-6	978	975	-	0.85	-	-	-	0.36	-	-
1-Octen-3-ol	3391-86-4	980	979	0.76	16.03		trace	-	0.36	-	4.51
NN (43,99,71,29,83)	-	985	-	-	0.17	-	-	-	-	-	-
Octan-3-one	106-68-3	986	986	4.80	-	-	-	-	-	-	3.96
6-Methyl-5-hepten-2-one	110-93-0	987	986	-	0.81	-	-	-	-	-	-
Hexanoic acid	142-62-1	988	990	-	-	0.32	-	-	-	-	-
2,3-Dehydro-1,8-cineol	66113-06-2	989	990	-	-	-	-	-	trace	-	-
Myrcene	123-35-3	990	991	-	-	-	-	2.37	-	0.81	1.61
2-Pentylfuran	3777-69-3	992	992	2.87	1.30	1.65	-	-	-	-	-
Mesitylene	108-67-8	992	993	-	-	-	1.93	-	-	-	-
NN (118,60,73,105,89)	-	995	-				-	-	-	-	-
3-Octanol	589-98-0	996	996	0.46	trace		-	-	-	-	-
(*E,Z*)-2.4-Heptadienal	4313-02-4	998	998	-	0.36	0.60	-	-	-	-	-
n-Decane	124-18-5	1000	1000	1.03	0.54	2.84	-	-	trace	-	-
Octanal	124-13-0	1002	1002	-	1.99	-	-	-	-	-	-
Mentha1,5,8-triene	21195-59-5	1003	1005	-	-	-	-	-	-	0.37	-
NN (56,84,29,41,55)	-	1004	-	0.53	-		-	-	-	-	-
α-Phellandrene	99-83-2	1004	1004	-	-	-	0.28	-	-	-	20.04
NN (mixture)	-	1005	-	-	-	-	-	0.44	-	-	-
3-Carene	13466-78-9	1009	1010	1.50	0.16	1.88	4.26	0.77	0.68	-	0.88
m-Methylanisole	100-84-5	1010	1012	-	-	-	0.93	-	-	-	-
(*E,E*)-2.4-Heptadienal	4313-03-5	1011	1012	-	0.21	-		-	-	-	-
Methyl-1,4-benzoquinone?	553-97-9	1014	-	0.19	-	-		-	-	-	-
α-Terpinene	99-86-5	1016	1015	-	-	-	-	0.69	-	-	-
1,2-Benzisoxazole?	271-95-4	1018	-	-	-	-	0.34	-	-	-	-
m-Cymene	535-77-3	1021	1021	-	-	-	2.90	-	-	-	-
p-Cymene	99-87-6	1022	1024	0.19	0.33	0.37	2.98	0.97	0.94	1.47	3.91
Limonene	138-86-3	1028	1028	2.19	0.51	0.48	2.26	1.17	0.96	2.41	8.73
1.8-Cineole	470-82-6	1031	1031	-	0.15	-	-	2.56	0.32	0.25	0.42
2-Ethylhexanol	107-76-7	1031	1032	0.57	-	-	0.51	-	-	-	-
Benzyl alcohol	100-51-6	1035	1033	trace	1.20	4.49	-	-	-	-	-
NN (83,69,55,56,140)	-	1037	-	-	-	-	-	-	0.32	-	-
o-Cymene	527-84-4	1037	1039	-	-	-	0.31	-	-	-	-
3-Octen-2-one	1669-44-9	1039	1039	-	1.04	-		-	-	-	-
β-Isophorone	471-01-2	1040	1044	-	-	-	-	-	0.02	-	-

Aromatics $C_{10}H_{14}$	-	1041	-	-	-	-	3.18	-	-	-	-
Salicyl aldehyde	90-02-8	1042	1041	-	-	1.05	-	-	-	trace	-
(*Z*)-β-Ocimene	3338-55-4	1043	1042	-	-	-	-	trace	-	-	-
Benzeneacetaldehyde	122-78-1	1045	1045		0.28	-	-	-	-	-	-
(*E*)-β-Ocimene	3779-61-1	1048	1048	-	0.59	0.56	-	5.49	-	-	-
(*E*)-2-Octenal	2548-87-0	1054	1057	-	1.28	-	-	-	-	-	-
γ-Terpinene	99-85-4	1059	1058	-	-	-	-	1.19	0.02	-	0.40
1,2-Cyclohexadienol?	-	1062	-	-	-	1.11	-	-	-	-	-
(*E*)-2-Octen-1-ol	18409-17-1	1067	1069	-	0.33	-	-	-		-	-
1-Octanol	111-87-5	1071	1070	trace	2.39	-	-	2.27	-	-	-
Dihydromyrcenol	18479-58-5	1072	1073	-	-	0.32	-	-	-	-	-
trans-Furanolinalool oxide	34995-77-2	1075	1073	3.02	0.22	-	0.88	-	4.69	-	-
(*E,E*)-Octa-3,5dien-2-one?	30086-02-3	1075	1083	-	-	-	-	-	-	-	0.52
m-Cymenene	1124-20-5	1082	1082	-	-	-	0.38	-	-	-	-
Terpinolene	586-62-9	1085	1086	-	-	-	-	0.50	-	-	-
cis-Furanolinalool oxide	5989-33-3	1088	1088	3.24	trace	-	trace	-	1.30	-	trace
1-Undecene	821-95-4	1091	1092	5.18	0.46	0.25	1.81	0.24	5.85	-	-
2-Nonanone	821-55-6		1094	-	0.40	-	-	0.29	-	-	-
Methyl benzoate	93-58-3	1095	1094	-	-	trace	-	-	-	-	-
NN (41,81,39,55,59)	-	1100	-	-	-	-	-	-	-	0.53	-
n-Undecane	1120-21-4	1100	1100	1.47	0.95	1.30	-	trace	trace	-	-
α-Pinene epoxide?	1686-14-2	1103	1097	-	-	-	-	-	-	4.86	-
Linalool	78-70-6	1103	1101	-	0.59	-	-	-	-	-	-
Nonanal	124-19-6	1107	1104	1.26	9.34	0.57	0.34	trace	-	-	0.57
keto-Pyranolinalool oxide	33933-72-1	1107	1108	0.40	-	-	-	-	2.72	-	-
α-Fenchol	14575-74-7	1113	1115	-	-	-	-	-	-	0.37	-
α-Campholenal	4501-48-0	1124	1126	-	-	-	-	-	-	0.44	-
2-Phenylethanol	60-12-8	1114	1112	0.33	1.52	0.27	-	trace	-	-	-
(*E*)-4,8-Dimethyl-1,3,7-nonatriene	51911-82-1	1117	1117	-	-	0.94	-	0.35	-	-	-
trans-3,4-Epoxycarane	N/A	1133	1134	-	-	-	0.56	-	-	-	-
trans-Pinocarveol	547-61-5	1137	1134	1.02	1.64	-	-	-	-	6.55	-
p-Menthan-8-ol?	498-81-7	1140	-	-	-	-	-	-	-	trace	-
Benzyl cyanide	140-29-4	1141	1139	-	-	-	-	trace	-	-	-
(*E*)-Verbenol	1820-09-3	1142	1140	-	-	-	-	-	-	9.31	-
Camphor	76-22-2	1144	1144	-	-	-	-	-	-	-	0.35
Veratrol	91-16-7	1152	1149	-	-	-	0.32	-	-	-	-
NN (43,125,167,95,69)	-	1152	-	-	-	-	-	-	0.31	-	-
(*E,Z*)-2,6-Nonadienal	557-48-2	1153	1155	-	1.15	-	-	-	-	-	-
Terpenoid $C_{10}H_{16}O$	-	1157	-	-	-	-	-	-	-	0.29	-
(*E*)-2-Nonenal	18829-56-6	1160	1160	trace	3.45	-	-	-	-	-	-
Pinocarvone	16812-40-1	1161	1160	0.59	-	-	-	-	-	0.65	-
Borneol	507-70-0	1162	1166	-	-	-	-	-	-	1.55	-
NN (101,56,83,55,126)	-	1167	-	-	-	-	-	-	0.25	-	-
NN (67,68,57,82,138)	-	1169	-	-	-	-	0.34	-	-	-	-
cis-Pyranolinalool oxide	14009-71-3	1169	1170	trace	0.35	-	-	-	-	-	-
NN (101,56,83,55,69)	-	1172	-	-	-	-	-	-	0.40	-	-
1-Nonanol	143-08-8	1173	1172	-	0.55	-	-	-	-	-	-
Carbonyl $C_{10}H_{14}O$?	-	1173	-	-	-	-	0.65	-	-	-	-
4-Terpineol	562-74-3	1173	1174	-	-	-	-	-	-	2.00	-

trans-Pyranolinalool oxide	41720-62-1	1175	1175	0.90	0.39	-	-	-	0.93	-	-
NN (152,154,139,122,66)	-	1178	-	0.51	-	-	-	-	-	-	-
NN (43,153,82,111,83)	-	1182	-	-	-	-	-	-	0.31	-	-
p-Cymen-8-ol	1197-01-9	1183	1184	-	-	-	-	-	-	1.56	-
α-Terpineol	98-55-5			-	-	-	-	-	-	4.32	-
1-Dodecene	112-41-4	1192	1192	0.29	0.45	0.19	-	-	-	-	-
NN (69,97,59,126,43)	-	1195	-	-	-	-	-	-	0.76	-	-
Myrtenol	515-00-4	1197	1196	0.94	3.80	-	-	-	-	5.59	-
$C_{10}H_{16}O_2$? (43,153,82,111,83…168)	-	1199	-	-	-	-	-	0.45	-	-	-
n-Dodecane	112-40-3	1200	1200	0.52	0.76	0.28	-	-	trace	-	-
NN (95,41,93,79,91,121)	-	1201		-	-	-	-	-	-	0.57	-
Verbenone	80-57-9	1206	1214	-	-	-	-	-	-	1.13	-
Decanal	112-31-2	1207	1206	0.22	1.27	trace	0.11	-	-	-	-
$C_{10}H_{18}O_2$? (MW 170)	-	1210	-	-	-	-	-	-	1.70	-	-
(*E,E*)-2,4-Nonadienal	5910-87-2	1214	1214	-	0.62	-	-	-	-	-	-
1,3,3-Trimethyl-2-oxabicyclo[2.2.2]octan-6-one	107598-08-3	1214	1217	-	-	-	-	-	1.83	-	-
(*E*)-Carveol	1197-07-5	1217	1219	-	-	-	-	-	-	1.16	-
2-Hydroxy-1,8-cineole	18679-48-6	1224	1224	-	-	-	-	-	0.17	-	-
Thymol methyl ether	1076-56-8	1236	1236	-	-	-	0.18	-	-	-	-
Myrtanol	514-99-8	1259	1257	1.30	1.27	-	-	-	-	-	-
(*E*)-2-Decenal	3913-81-3	1262	1263	-	0.82	-	-	-	-	-	-
Nonanoic acid	112-05-0	1276	1274	-	0.38	trace	-	-	-	-	-
NN (123,107,95,192,121)	-	1282	-	-	-	-	-	-	0.21	-	-
Bornyl acetate	76-49-3	1286	1287	0.29	0.20	trace	0.18	-	trace	8.18	-
cis-2-*tert*-Butyl cyclohexanol acetate	20298-69-5	1292	1295	-	-	-	0.43	-	0.17	-	-
1-Tridecene	2437-56-1	1294	1292	0.61	0.88	0.36	-	-	-	-	-
Unknown sesquiterpene-1	-	*1298*	-		*1.30*	-	-	-	*1.05*	-	-
n-Tridecane	629-50-5	1300	1300	1.89	0.62	0.33	-	-	trace	-	0.40
Unknown sesquiterpene-2	-	*1307*	-	-	*2.57*	-	*0.11*	-	*3.03*	-	*trace*
Undecanal	112-44-7	1311	1309		0.17	0.25	-	-	-	-	-
NN (125,43,126,109,107)	-	1318	-	-	-	-	0.14	-	-	-	-
Unknown sesquiterpene-3	-	*1318*	-	-	*0.68*	-	-	-	*0.29*	-	-
NN (57,85,99,71,113)	-	1320	-	-	-	-	0.18	-	-	-	-
NN (123,165,81,95,43)	-	1324	-	-	-	-	0.14	-	0.18	-	-
Unknown sesquiterpene-4	-	*1324*	-	-	*0.36*	-	-	-	-	-	-
NN (159,117,131,174,91)	-	1326	-	-	0.31	-	-	-	0.31	-	0.36
Unknown sesquiterpene-5	-	1345	-	-	-	-	0.38	-	1.54	-	-
α-Cubebene	17699-14-5	1350	1351	-	0.91	0.18	0.97-	-		-	-
Sesquiterpene $C_{15}H_{24}$	-	1351	-	-	-	-	-	-	-	0.50	1.45
α-Terpenyl acetate	80-26-2	1354	1351	-	-	-	-	-	-	2.95	
Eugenol	97-53-0	1358	1358	-	0.33	-	-	-	-	-	
Unknown sesquiterpene-6	-	*1359*	-	-	*0.52*	-	-	-	-	-	
Cyclosativene	22469-52-9	1367	1368	0.86	0.35	-	-	-	0.38	-	0.38
Longocyclene	1137-12-8	1367	1370	-	-	-	0.69	-	0.73	-	-
α-Ylangene	14912-44-8	1372	1372	0.65	2.82	trace	0.48	-	4.87	1.20	4.38
α-Copaene	3856-25-5	1376	1376	1.51	6.44	0.80	2.48	-	11.18	1.14	8.99
7-Cubebene?	N/A	1377	1381	-	-	-	-	-	0.32	-	-

Daucene	16661-00-0	1379	1381	-	-	-	trace	-	-	-	-
β-Bourbonene	5208-59-3	1388	1387	0.44	0.93	0.45	2.10	-	1.18	-	0.27
β-Cubebene	13744-15-5	1389	1392	-	-	-	0.22	-	-	-	-
β-Elemene	515-13-9	1391	1392	-	-	-	0.68	-	0.21	-	-
Sesquiterpene $C_{15}H_{24}$	-	1392	-	-	-	-	-	-	0.26	0.25	-
1-Tetradecene	1120-36-1	1392	1392	-	-	0.23	-	-	-	-	-
n-Tetradecane	629-59-4	1400	1400	trace	0.47	0.48	-	trace	-	-	-
Sesquiterpene $C_{15}H_{24}$	-	1400	-	-	-	-	-	-	2.29	-	-
Longifolene	475-20-7	1402	1404	-	-	-	1.02	-	-	10.43	6.27
α-Barbatene	N/A	1406	1410	0.19	-	-	-	-	-	-	-
Dodecanal	112-54-9	1409	1409	-	-	trace	-	-	-	-	-
(Z)-α-Bergamotene	13474-59-4	1411	1416	-	-	-	-	-	0.30	-	-
Sesquiterpene $C_{15}H_{24}$	-	1412	-				0.27	-	-	-	-
β-Caryophyllene	87-44-5	1416	1417	0.28	0.63	0.87	2.77	1.04	2.34	-	0.72
Sesquiterpene $C_{15}H_{24}$	-	1423	-	-	-	-	-	-	-	0.93	-
β-Copaene	18252-44-3	1426	1432	0.12	0.32	-	0.40	-	0.97	-	-
(*E*)-α-Bergamotene	13474-59-4	1435	1436	-	-	-	-	-	0.30		-
NN (173,161,131,145,105)	-	1429	-	-	-	-	-	-	-	-	0.45
β-Gurjunene	17334-55-3	1429	1434	-	-	-	0.19	-	-	-	-
Octyl isopentanoate	7786-58-5	1437	1439	0.37	-	-	-	-	-	-	-
β-Barbatene	72346-55-5	1439	1443	-	-	-	-	-	0.23	-	-
Guaia-6,9-diene	36577-33-0	1440	1445	0.74	3.10	-	0.57	-	5.99	-	0.55
Aromadendrene	489-39-4	1441	1440	0.37	-	0.35	0.72	-	1.40	-	1.44
NN (194,151,180,207,193)	-	1444	-	-	-	-	0.57	-	-	-	-
Sesquiterpene $C_{15}H_{24}$	-	1451	-	0.31	0.82	-	0.32	-	-	0.26	-
Sesquiterpene $C_{15}H_{24}$	-	1452	-	-	1.37	-	-	-	-	-	-
α-Humulene	6753-98-6	1454	1454	-	0.28	-	0.46	trace	0.59	-	0.39
Selina-4(15),6-diene	N/A	1455	1454	-	-	-	-	-	2.06	-	-
Sesquiterpene $C_{15}H_{24}$	-	1457	-	-	-	-	-	-	-	0.26	2.69
4-Methyl tetradecane	25117-24-2	1458	1459	-		-	0.50	-	-	-	-
Sesquiterpenoid $C_{15}H_{24}$	-	1461	-	-	-	-	0.33	-	-	-	-
Alloaromadendrene	25246-27-9	1463	1461	-	1.16	-	0.90	-	1.86	-	2.05
NN (127,43,107,136,122)	-	1464	-	-	-	-	-	-	-	-	0.49
Sesquiterpenoid $C_{15}H_{24}$	-	1468	-	-	-	-	0.33	-	0.67	-	-
NN (137,215,59,77,237)	-	1469	-	-	-	0.65	-	-		-	-
γ-Muurolene	30021-74-0	1472	1476	-	0.62	0.23	1.40	-	1.69	-	1.10
β-Chamigrene	18431-82-9	1474	1475	-	0.24	0.16	0.88	-	-	-	-
α-Amorphene	483-75-0	1479	1480	-	0.33	-		-	0.50	1.55	0.43
Germacrene D	23986-74-5	1480	1480	-	-	-	-	trace	-	-	-
β-Selinene	17066-67-0	1482	1485	-	-	trace	1.32	-	-	-	0.49
Aristolochene	26620-71-3	1482	1486	-	-	-	-	-	1.84	-	-
α-Selinene	473-13-2	1496	1496	-	-	trace	1.53	-	-	-	-
(*Z,E*)-α-Farnesene	26560-14-5	1498	1496	-	-	-	-	0.79	0.61	-	-
α-Muurolene	10208-80-7	1499	1500	-	-	0.29	1.11	-	-	-	0.70
n-Pentadecane	629-62-9	1500	1500	0.12	0.46	0.20	-	trace	-	-	-
(*E,E*)-α-Farnesene	502-61-4	1511	1509	-	1.53	1.20	-	21.67	-	-	-
γ-Cadinene	39029-41-9	1513	1517	-	0.36	0.43	1.82	-	0.79	-	0.70
δ-Cadinene	483-76-1	1524	1524	-	0.41	0.37	2.37	trace	1.20	-	1.11
Cesquiterpene $C_{15}H_{24}$	-	1527	-	-	-	-	-	0.54	-	-	-

(*E*)-Cadina-1,4-diene	38758-02-0	1531	1536	-	-	-	-	-	0.26	-	-
C15H30 (83,71,69,43,210)	-	1535	-	-	-	-	-	-	1.31	-	-
α-Cadinene	24406-05-1	1540	1540	-	-	-	0.29	-	-	-	
α-Calocorene	21391-99-1	1544	1544	-	-	-	0.27	--	-	-	
(*E*)-Dendralasine	23262-34-2	1580	1575	-	-	0.27	-	0.78	-	-	
Caryophyllene oxide	1139-30-6	1581	1582	-	0.14	-	-	-	-	5.04	
Salvial-4(14)-en-1-one	N/A	1592	1598	-	0.12	-	-	-	-	-	0.29
n-Hexadecane	544-76-3	1600	1600	-	0.13	0.25	-	-	-	-	-
Sesquiterpene $C_{15}H_{24}$	-	1600	-	-	-	-	-	079	-	-	-
Humulene epoxyde II	19888-34-7	1609	1609	-	-	-	-	-	-	0.53	-
Diter (91,257,147,105,109)	-	1635	-	-	-	-	0.19	-	-	-	-
n-Heptadecane	629-78-7	1700	1700	-	0.08	-	-	-	-	-	-
Hexafarnesyl acetone	505-69-2	1845	1846	-	-	0.28	0.34	-	-	-	-
n-Nonadecane	629-92-5	1900	1900	-	trace	-	-	-	-	-	-
Rumuene? $C_{20}H_{32}$	1686-67-5	1946	1930	-	1.37	-	-	-	-	-	-
Diterpene (257,91…272)	-		-	-	0.76	-	-	-	-	-	-

* - not found; ** below 0.01% of TIC; *** the identification of the corresponding compound is considered preliminary.

Table S2. Chemical composition of methanol extracts of fallen inflorescences of some forest-forming deciduous trees. 1 – *C. betulus,* 2 – *P. tremula,* 3 – *A. platanoides,* 4 – *B. pendula,* 5 – *S. fragilis*

Compound (TMS)	CAS	RI^{Calk}	RI^{DB}	1	2	3	4	5
2,3-Butanediol, isomer 1, di-TMS	53274-85-4	1041	1042	-*	-	0,05	-	
2,3-Butanediol, isomer 2, di-TMS	N/A	1049	1050	-	-	0,08	-	
Lactic acid, di-TMS	17596-96-2	1076	1073	-	-	0,05	-	
NN (89,73,175,75, 59)	-	1080	-	-	-	0,16	-	
Glycolic acid, di-TMS	33581-77-0	1084	1083	0.04	0.12	0,03	-	
Valine, mono-TMS	7480-78-6	1090	1089	-	-	trace	-	
Alanine, N,O-di-TMS	2899-44-7	1113	1114	-	-	0,14	-	
Benzyl alcohol, TMS	14642-79-6	1155	1156	-	trace**	-	-	
Proline, mono-TMS	N/A	1174	1174	-	trace	0,14	-	
Isoleucine, mono-OTMS	N/A	1180	1179	-	trace	-	-	
Malonic acid	18457-04-0	1217	1216	-	--	0,06	-	
Valine, di-TMS	7364-44-5	1227	1227	-		0,23	-	
Benzoic acid, TMS	2078-12-8	1246	1246	-	0.37	-	-	
Urea, di-TMS	18297-63-7	1253	1255	-	-	0,15	-	
1,2-Hihydroxycyclohexane-1, di-TMS	39789-21-4	1262	1263	-	trace	-	-	
Serine, O,O-di-TMS	70125-39-2	1268	1265	-	trace	0,08	-	
1,2-Hihydroxycyclohexane-2, di-TMS	N/A	1277	1276	-	trace	-	-	
Leucine, N,O-di-TMS	15984-97-1	1286	1284	-	0.54	0,12	-	
H_3PO_4, tri-TMS	10497-05-9	1291	1289	-	0.46	0,62	0.15	
Glycerol, tri-TMS	6787-10-6	1295	1294	-	1.24	2,01	0.66	
Proline, di-TMS	7364-47-8	1305	1303	-	0.30	0,83	-	
Isoleucine, di-TMS	7483-92-3	1308	1308	-	0.56	-	-	
γ-Aminobutyric acid, di-TMS	39538-11-8	1310	1310	-	0.15	-	-	
Pyrocatechol, di-TMS	5075-52-5	1322	1323	-	0.12	-	-	
Succinic acid, di-TMS	40309-57-7	1326	1324	0.33	0.88	0,19	0.40	
Glyceric acid, tri-TMS	38191-87-6	1351	1348	0.17	0.35	0,26	0.08	

Fumaric acid, di-TMS	17962-03-7	1359	1355	trace	trace	0,05	-	
Serine, N,O,O-tri-TMS	64625-17-8	1374	1370	-	0.17	0,16	-	
4-Hydroxybenzaldehyde, TMS	1012-12-0	1373	1373	0.05	-	-	-	
Dihydro-2(3H)-furanone <(*Z*)-3,4-dihydroxy->, di-TMS	55220-75-2	1386	1384	0.16	-	0,04	-	
Threonine, tri-TMS	7537-02-2	1408	1406	-	0.24	0,17	-	
NN	-	1407	-	0.12	-	-	-	
NN	-	1409	-	0.42	-	-	-	
Salicyl alcohol, di-TMS	18544-92-8	1444	1445	-	0.16	-	-	
2-Pyrrolidone-5-carboxylic acid, TMS	N/A	1505	1509	0.51	-	-	-	
Malic acid, tri-TMS	38166-11-9	1512	1510	4.85	2.65	1,04	3.29	
Pyroglutamic acid, di-TMS	30274-77-2	1532	1530	0.61	0.21	0,26	-	
Threitol, tetra-TMSTMS	32381-52-5	1539	1540	0.06	0.21	0,16	0.26	
GABA, tri-TMS	39508-23-1	1540	1541	-	0.31	0,16	-	
NN	-	1560	-	-	-	-	0.13	
Butyric acid, 2,3,4-trihydroxy-, tetra-TMS	N/A	1578	1575	-	0.91	-	-	
Butyric acid, 2,3,4-trihydroxy-, tetra-TMS	38191-88-7	1593	1597	0.72	0.14	0,92	0.12	
Asparagine, N2,O-di-TMS	N/A	1606	-	-	-	0,04	-	
NN	-	1630		-	-	-	0.18	
L-Tartaric acid, tetra-TMS	N/A	1630	-	1.36		0,43	-	
4-Hydroxybenzoic acid, di-TMS	2078-13-9	1635	1636	-	0.07	-	-	
Phenylalanine, di-TMS	2199-52-7	1640	1635	-	0.07	0,20	-	
Glutamine, N,O,O-tri-TMS	15985-07-6	1642	1642	-	0.22	0,09	-	
Arabinose, tetra-TMS	1768-95-2	1647	1648	0.07	-	0,07	0.09	
Rhamnose, tetra-TMS	19127-15-2	1660	1661	0.12	-	0,06	0.07	
Xylofuranose, tetra-TMS, isomer 1	N/A	1668	1670	-	-	0,06	-	
α-Ribofuranose, tetra-TMS	56271-69-3	1678	1678	-	-	0,05	0.11	
Xylofuranose, tetra-TMS, isomer 2	N/A	1678	1680	0.08	-	0,11	0.09	
3-Hydroxyadipic acid, tri-TMS	73105-00-7	1692	1698	trace	-	-	-	
Asparagine, tri-O,O',N-TMS	N/A	1690	1691	-	0.61	0,34	-	
NN	-	1696	-	0.19	-	-	-	
Fucose, tetra-TMS	N/A	1700	1699	-	-	0,11	0.07	
Pentose, TMS (m/z 204)	-	1720	-	0.17	-	0,11	0.08	
Xylopyranose, tetra-TMS	18623-22-8	1736	1740	0.23	-	0,56	0.94	
Xylitol, penta-TMS	14199-72-5	1745	1745	-	-	0,06	-	
Pentitol, penta-TMS	-	1757	-	0.25	-	0,29	0.45	
Ribitol, penta-TMS	32381-53-6	1766	1761	-	-	0,05	-	
Arabinitol, penta-TMS	25138-28-7	1776	1772	0.24	0.34	0,03	-	
Lyxofuranose, tetra-TMS	N/A	1778	1770	-	0.52	-	0.72	
Pentonic acid, TMS	-	1788	-	-		0,09	-	
β-Xylopyranose, tetra-TMS	N/A	1792	1792	-		0,63	1.21	
α-Glycerophosphoric acid, tetra-TMS	31038-11-6	1797	1797	0.21		0,13	-	
Glutamine, N,N',O-tri-TMS	70591-28-5	1795	1798	-	0.93	-	-	
α-Glycerphosphoric acid, tetra-TMS	31038-11-6	1799	1797	-	0.10	-	-	
β- Xylofuranose, tetra-TMS?***	N/A	1800	1800	0.25	-	0,26	0.30	
NN	-	1804	-	0.18	-	0,17	-	
Azelaic acid, di-TMS	17906-08-0	1808	1808	0.10	-	-	-	
α-Methylfuranoside, tetra-TMS	30788-71-7	1813	1817	0.48	-	0,12	0.47	
L-Fucitol, penta-TMS?	N/A	1817	-	-	-	-	0.08	
NN	-	1828	-	0.65	0.15	0,46	-	
α-Methylmannopyranoside, tetra-TMS	1769-06-8	1828	1822	-	-	-	0.68	

Hexapyranoside, TMS	-	1831	-	-	-	-	0.09	
Inositol, deoxy-, penta-TMS	114656-62-1	1833	1834	0.53	-	-	-	
Protocatechuic acid, tri-TMS	2347-40-2	1836	1836	0.06	-	0,16	0.07	
α-Fructofuranose, penta-TMS	N/A	1845	1850	6.90	2.85	6,16	5.91	
β-Fructofuranose, penta-TMS	N/A	1853	1854	5.26	8.43	7,38	5.44	
NN (73,368,369,370)	-	1861	-	-	-	-	0.70	
α-Galactofuranose, penta-TMS	55529-72-1	1865	1860	-	-	2,64	-	
Pinitol, penta-TMS	N/A	1872	1872	-	-	0,88	-	
Hexafuranoside, TMS	-	1875	-	-	0.34	-	-	
Hexapyranoside, TMS	-	1877	-	-	-	-	1.41	
α-Fructopyranose, penta-TMS	N/A	1888	1889	1.24	0.32	0,75	0.59	
α-Glucofuranose	66807-66-7	1892	1889	1.22	0.56	1,00	0.95	
Hexafuranose, penta-TMS	-	1896	-	-	1.04	1,01	0.62	
Galactopyranose, penta-TMS	32166-80-6	1899	1900	-	0.51	-	-	
NN (73,368,204,191)	-	1902	-	-	-	-	1.84	
Cyclohexanepentol, penta-TMS	N/A	1915	1907	6.76	-	13,76	-	
2-Amino-2-deoxyglucose, tetra-TMS	74978-27-1	1912	19909	1.17	-	-	-	
Methygallate, tri-TMS	N/A	1920	1921	1.40	-	3,16	-	
α-Glucopyranose, penta-TMS	3327-61-5	1932	1932	11.34	9.50	8,24	9.57	
β-Mannopyranoside, TMS	55529-69-6	1942	1943	0.52		0,22	0.52	
p-Coumaric acid, di-TMS	10517-30-3	1946	1947	-	1.18	-	-	
β-Talopyranose, penta-TMS	N/A	1950	1949	-	-	0,57	-	
Furanoside. (73,217,218,147,103)	-	1964	-	-	0.17	-	-	
Aminocarbohydrate, TMS	-	1967	-	0.07	-	-	-	
Mannitol, hexa-TMS	14317-07-8	1973	1972	0.41	0.41	0,13	0.28	
Glucitol, hexa-TMS	14199-80-5	1979	1976	-	0.14	0,87	-	
Ascorbic acid, tetra-TMS	55517-56-1	1980	1980	0.35	-	-	-	
Hexapyranoside, TMS	-	1983	-	-	-	-	0.41	
Altitol, hexa-TMS	N/A	1983	1984	-	-	0,29	-	
Gallic acid, tetra-TMS	2078-17-3	1988	1985	3.24	0.19	3,39	0.46	
Pinitol, penta-TMS	N/A	1996	1996	1.04	-	0,18	-	
chiro-Inositol, hexa-TMS	29412-25-7	2001	1997	3.62	trace	0,19	-	
NN, TMS	-	2002	-	-	-	-	0.36	
NN	-	2008	-	0.22	-	0,19	-	
Hexafuranose, TMS	-	2028	-	-	-	0,14	0.47	
β-Glucopyranose, penta-TMS	2775-90-8	2030	2032	7.86	10.85	8,79	8.04	
Gluconic acid, hexa-TMS	34290-52-3	2044	2046	-	0.29	0,21	-	
Hexadecanoic acid, TMS		2052	2052	0.42	0.48	0,44	0.27	
Hexapyranoside, TMS	-	2079	-	0.21	-	0,35	0.98	
(*E*)-Ferulic acid, do-TMS	10517-09-6	2101	2101	-	0.32	-	-	
Pyranoside, TMS	-	2108	-	-	-	-	0.17	
Methoxycyclohexanepentol, penta-TMS	N/A	2114	2113	-	-	-	0.15	
myo-Inositol, hexa-TMS	2582-79-8	2128	2131	2.25	1.77	2,68	2.05	
N-Acetylglucosamine, tetra-TMS	31980-72-0	2143	2139	0.14	-	-	0.19	
N-Acetylamino carbohydrate, tetra-TMS	N/A	2151	-	-	-	-	0.08	
(*E*)-Caffeic acid, tri-TMS	10586-03-5	2152	2154	-	trace	-	-	
N-Acetylcarbohydrate, TMS	-	2154	-	0.07	-	-	0.12	
Hexapyranoside, TMS	-	2159	-	-	0.09	-	-	
Hexapyranoside, TMS	-	2182	-	-	--	0,28	-	
Hexapyranoside, TMS	-	2192	-	-	-	1,23	-	
NN		22o3	-	-	-	0,08	-	

Linoleic acid, TMS	56259-07-5	2215	2215	0.23	0.46	0,28	0.30	
α-Linolenic (& oleic acid), TMS	97844-13-8	2222	2218	0.20	0.54	0,39	0.17	
Tryptophane, tri-TMS	55429-28-2	2236	2233	-	0.19	-	-	
Carbohydrate, TMS	-	2244	-	-	-	-	0.29	
Hexapyranoside, TMS	-	2246	-	0.20	0.35	0,44	0.49	
Stearic acid, TMS	18748-91-9	2249	2250	0.14	trace	0,13	trace	
Hexapyranoside, TMS	-	2258	-	0.27	-	0,16	0.34	
myo-Inositol phosphate, penta-TMS	55518-06-4	2258	2260	-	0.26	-	-	
Carbohydrate, TMS	-	2261	-	-	-	-	0.47	
Carbohydrate, TMS (73=204,147,217,205)	-	2308	-	-	0.28	-	0.18	
Carbohydrate/glycoside, TMS	-	2346	-	0.12		0,14	-	
Carbohydrate/glycoside, TMS	-	2362	-	-	-	-	0.09	
Carbohydrate/glycoside, TMS				-	-	-		
Carbohydrate/glycoside, TMS	-	2370	-	-		0,10	-	
Galactopyranoside, 2-Oglycerol-α-D-, hexa-TMS	N/A	2374	2375	-	0.31	-	-	
Carbohydrate/glycoside, TMS	-	2378	-	-	-	-	0.07	
Carbohydrate/glycoside, TMS	-	2380	-	-	-	-	0.08	
Carbohydrate, acetylamino-? TMS	-	2404	-	-	-	-	0.07	
Carbohydrate/glycoside, TMS	-	2413	-	-	-	0,11	0.15	
Carbohydrate/glycoside, TMS	-	2421	-	-	-	-	0.11	
Glucoside, TMS (204,73,**91**,217)	-	2439	-	0.28	0.47	0,49	0.38	
Carbohydrate/glycoside	-	2446	-	-	-	-	0.25	
NN	-	2453	-	-	0.48	0,21	0.36	
Uridine, triTMS	10457-16-6	2469	2468	0.15	-	0,13	0.08	
Glycoside, TMS? (204,73,**105**,205)	-	2549	-	-	-	0,12	-	
Pyrocatechol β-D-glucopyranoside, penta-TMS	N/A	2493	2491	-	0.28	-	-	
n-Pentacosane	629-99-2	2500	2500	-	-	-	0.14	
Carbohydrate/glycoside, TMS	-	2524	-	-	-	-	0.08	
Carbohydrate, TMS (73,204,217,147,287)	-	2532	-	-	0.15	-	-	
1-Dodecanol, TMS	42449-18-3	2558	2557	-	-	-	0.30	
Salicin, penta-TMS	N/A	2578	2580	-	2.75	-	-	
Carbohydrate/glycoside, TMS	-	2585	-	-	-	0,13	0.86	
Carbohydrate/glycoside, TMS	-	2591	-	-	-	0,06	0.09	
Carbohydrate/glycoside, TMS	-	2618	-	-	0.22	0,31	0.19	
NN (74,440,369,441,281)	-	2628	-	-	-	-	0.10	
Docosanoic acid, TMS + arbutin, penta-TMS	74367-36-5	2647	2647	-	-	-	0.10	
Carbohydrate/glycoside, TMS	-	2659	-	-	-	-	0.16	
Carbohydrate/glycoside, TMS	-	2664	-	-	-	0,29	-	
Adenosine riboside, tetra-TMS	53294-33-0	2672	2672	-	0.36	0,17	0.31	
NN	-	2681	-	-	0.33	0,19	0.17	
NN	-	2688	-	-	0.17	-	-	
Disaccharide, TMS	-	2696	-	-	-	0,17	-	
Xylobiose, hexa-TMS	N/A	2698	2694	-	-	-	0.57	
Disaccharide, TMS	-	2699	-	-	-	0,50	-	
n-Heptocosane	593-49-7	2700	2700	-	0.22	-	trace	
Sucrose, octa-TMS	19159-25-2	2718	2714	3.93	12.85	6,23	6.78	
Maltose, octa-TMS, isomer 1	33428-94-3	2748	2746	-	-	0,15	0.37	
Cellobiose, octa-TMS, isomer 1	56145-25-6	2764	2762	0.13	-	0,22	0.39	
Turanose, octa-TMS	N/A	2793	2793	0.15	-	0,18	0.10	

Maltose, TMS, isomer 2	N/A	2803	2800	-	0.30	0,14	0.08	
Palatinose, octa-TMS, isomer 1	N/A	2814	2818	-	-	0,18	-	
Disaccharide/glycoside, TMS	-	2822	-	2.85	-	0,13	-	
Salidroside, penta-TMS	1036766-19-4	2832	2828	-	-	-	1.86	
Glycoside (204,73,193,192,147)	-	2835	-	5.15	-	-	-	
Disaccharide (204,73,217,361,169)	-	2850	-	-	0.20	-	-	
Disaccharide (204,73,217,191,205)	-	2859	-	-	0.27	-	-	
Laminaribiose, octa-TMS, anomer 1	N/A	2862	2864	-	-	0,48	-	
Disaccharide/glycoside, TMS	-	2873	-	-	-	0,10	-	
Laminaribiose, octa-TMS, anomer 2	N/A	2889	2891	-	-	1,35	-	
Disaccharide, TMS	-	2890	-	-	0.33	-	-	
Glycoside, TMS	-	2897	-	-	-	0.94	-	
NN (73,395,396,397,147)	-	2900	-	-	-	-	1.29	
epi-Catechin, penta-TMS	N/A	2900	2907	-	-	-	0.79	
p-Coumaroylquinate, 5-TMS	-	2909	-	1.09	-	0,26	-	
Disaccharide, TMS (204,73,205,217,191)	-	2909	-	-	0.37	-	-	
Vanillic acid 4-β-glucoside, penta-TMS	N/A	2926	-	-	-	-	0.43	
Catechine, penta-TMS	91701-26-7	2936	2936	0.78	0.27	0,20	0.79	
NN (**369**,73,217,370,147)	-	2950	-	-	-	0,26	-	
NN (73,356,458,147)	-	2950	-	-	-	-	0.39	
Disaccharide/glycoside, TMS	-	2959	-	0.42	-	1,00	-	
NN (**369**,73,370,371,147)	-	2966	-	0.31	-	-	-	
NN (297,73,179,298)	-	2972	-	-	-	-	0.46	
Glycoside? (73,204,456,355)	-	2989	-	-	-	-	0.69	
NN	-	3003	-	0.53	-	-	-	
NN	-	3018	-	1.31	-	-	0.27	
Disaccharide, TMS	-	3045	-	0.28	-	-	0.40	
NN (**369**,73,370,217,371)	-	3050	-	0.12	-	-	-	
Apigenin, 7, 4'-di-TMS	N/A	3082	3087	-	0.45	-	-	
NN (**369**,370,73,371,281)	-	3101	-	0.29	-	1,36	-	
Glycoside, TMS (204,73,205,221,193)	-	3107	-	-	-	-	2.93	
Glycoside (105,73,393,106,147)	-	3112	-	-	1.55	-	-	
Kaempherol, tetra-TMS	N/A	3115	3115	-	-	0,07	-	
p-Coumaroylquinate, penta-TMS	N/A	3123	3123	0.21	-	0,07	0.19-	
Syringin, penta-TMS?	N/A	3140	3154	-	0.20	-	-	
Apigenin, tri-TMS	N/A	3158	3159	-	0.17	-	-	
Glicoside (191, 204,73,91,217)	-	3177	-	-	0.34	-	-	
Chlorogenic acid, hexa-TMS	N/A	3186	3183	0.24	0.30	-	0.43	
NN (**369**,370,73,371,281)	-	3205	-	0.12	-	0,93	-	
Quercetin, penta-TMS	4067-66-7	3210	3213	0.09	-	-	0.60	
Disaccharide/glycoside, TMS	-	3234	-	0.08	0.43	0,14	-	
Glucoside (204,217,73,105,205)	-	3242	-	-	-	-	0.21	
Cryptochlorogenic acid, hexa-TMS	N/A	3256	3253	0.09	-	-	-	
Neochlorogenic acid	N/A	3273	3268	0.19	-	-	-	
Glucoside, TMS	-	3274	-	-	-	-	0.24	
Stigmasterol, TMS	14030-29-6	3290	3285	0.05	-	-	-	
NN	-	3294	-	-	-	0,05	-	
Glycoside, TMS (73,361,1799,217,147)	-	3297	-	-	2.65	-	-	
Glycoside, TMS (73,361,105,193,217)	-	3323	-	-	0.21	-	-	
Ellagic acid, tetra-TMS	N/A	3329	3329	0.45		0,05	-	
NN (255,73,361,254,256)	-	3334	-	-	6.13	-	-	

β-Amyrin + β-sitosterol (mixture)		3347	3348	-	-	0,37	-	
β-Sitosterol, TMS	2625-46-9	3349	3345	0.17	0.24	-	0.37	
Disaccharide/glycoside	-	3362	-	0.07	-	-	-	
Ester (57,313,71,97,83)	-	3391	-	0.65	-	-	1.03	
Raffinosa, undeca-TMS	N/A	3503	3505	-	0.67	-	0.22	
1-Kestose, undeca-TMS	N/A	3517	3517	-	0.33	0,29	-	
Glycoside, 4-OH-phenylethyl-	-	3533	-	0.06	-	-	-	
Erlose, undeca-TMS	N/A	3549	3548	-	-	0,03	-	
Glycoside, TMS	-	3683	-	-	-	-	0.10	
NN (73,**368**,245,348,369)	-	3692	-	-	-	-	0.18	
Glucoside with 2-(4-hydroxyphenyl)-ethyl-moiety	-	3705	-	-	-	-	0.18	
Glicoside, TMS (**369**,73,370,204,281)	-	3707	-	0.12	-	-	-	
Glycoside (73,**368**,36,259)	-	3736	-	-	-	-	0.11	
Kaempherol 3-β-O-galactoside, TMS	N/A	3746	3740	-	-	0,51	-	
Kaempherol 3-β-glucopyranoside, hepta-TMS	N/A	3761	3754	-	-	0,46	-	
NN (73,209,217,117,147)	-	3769	-	-	-	-	0.08	
Arabinopyranoside (73,245,348,518)	-	3793	-	0.13	-	-	-	
Quercetin glycoside, TMS	-	3806	-	-	-	0,16	-	
Glycoside, TMS	-	3807	-	-	-	-	0.55	
Quercetin 3-galactoside, octa-TMS	N/A	3823	3826	-	-	0,18	-	
Quercetin 3-O-glucoside, octa-TMS	N/A	3836	3836	-	-	0,08	-	
NN (131,73,348,215,368)	-	3850	-	-	-	-	0.21	
Tremuloidin,tetra-TMS	N/A	3861	3861	-	0.66	-	-	
Catechin-7-O-glucoside, octa-TMS	N/A	3866	3860	-	-	-	7.18	
Glicoside, TMS (**369**,73,370,204,281)	-	3872	-	0.12	-	-	-	
Triterpenoid (216, 203,161,189,95)	-	3872	-	-	-	0,44	-	
Glicoside, TMS (73,**369,**204,277,370)	-	3878	-	0.55	-	-	-	
Glicoside, TMS (105,255,254,73,256)	-	3879	-	-	5.19	-	0.42	
Glycoside	-	3887	-	0.07	-	-	-	
Tremuloidin-like TMS	-	3897	-	-	0.54	-	-	
Quercetin 3-α-L-arabinopyranoside, hepta-TMS	N/A	3918	-	-	-	-	1.43	
Naringenin 7-O-glucoside, hexa-TMS?	-	3927	3914	0.40	-	0,11	-	
Glycoside with quercetine moiety	-	3931	-	-	-	-	2.08	
Ester (docosanoate?)	-	4096	-	1.29	-	-	1.95	
β-Sitosterol-β-D-glucoside, tetra-TMS	N/A	-	-	1.41	0.87	1,27	-	
Procyanidin B1, deca-TMS	N/A	-	4234	-	-	-	0.75	

* - not found; ** below 0.01% of TIC; *** the identification of the corresponding compound is considered preliminary.

Figure S1. Mass spectra of unidentified $C_{15}H_{24}$ sesquiterpenes (absent in available databases) in the composition of VOCs in hornbeam inflorescence litter

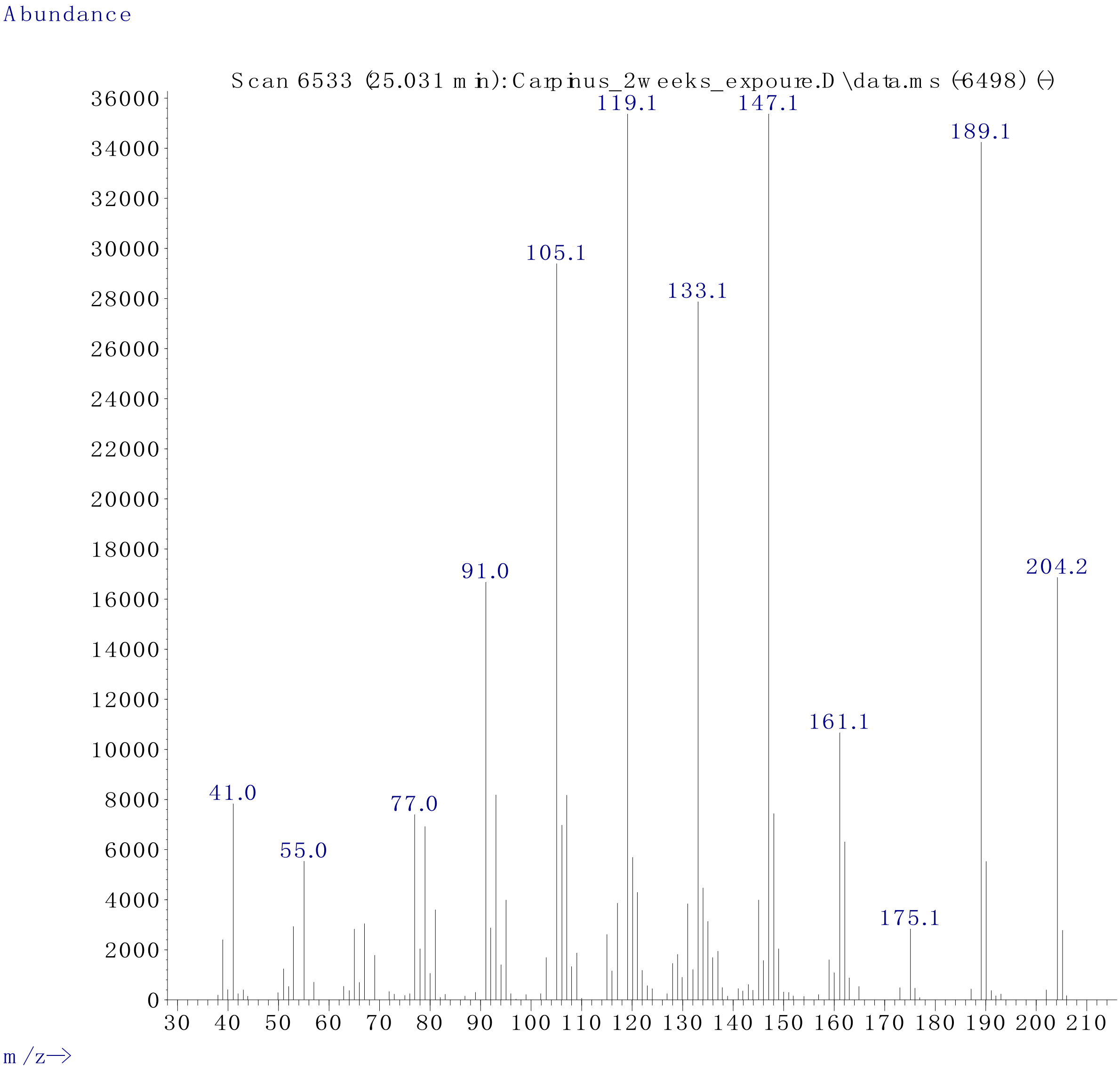

Unknown sesquiterpene-1, RI = 1298

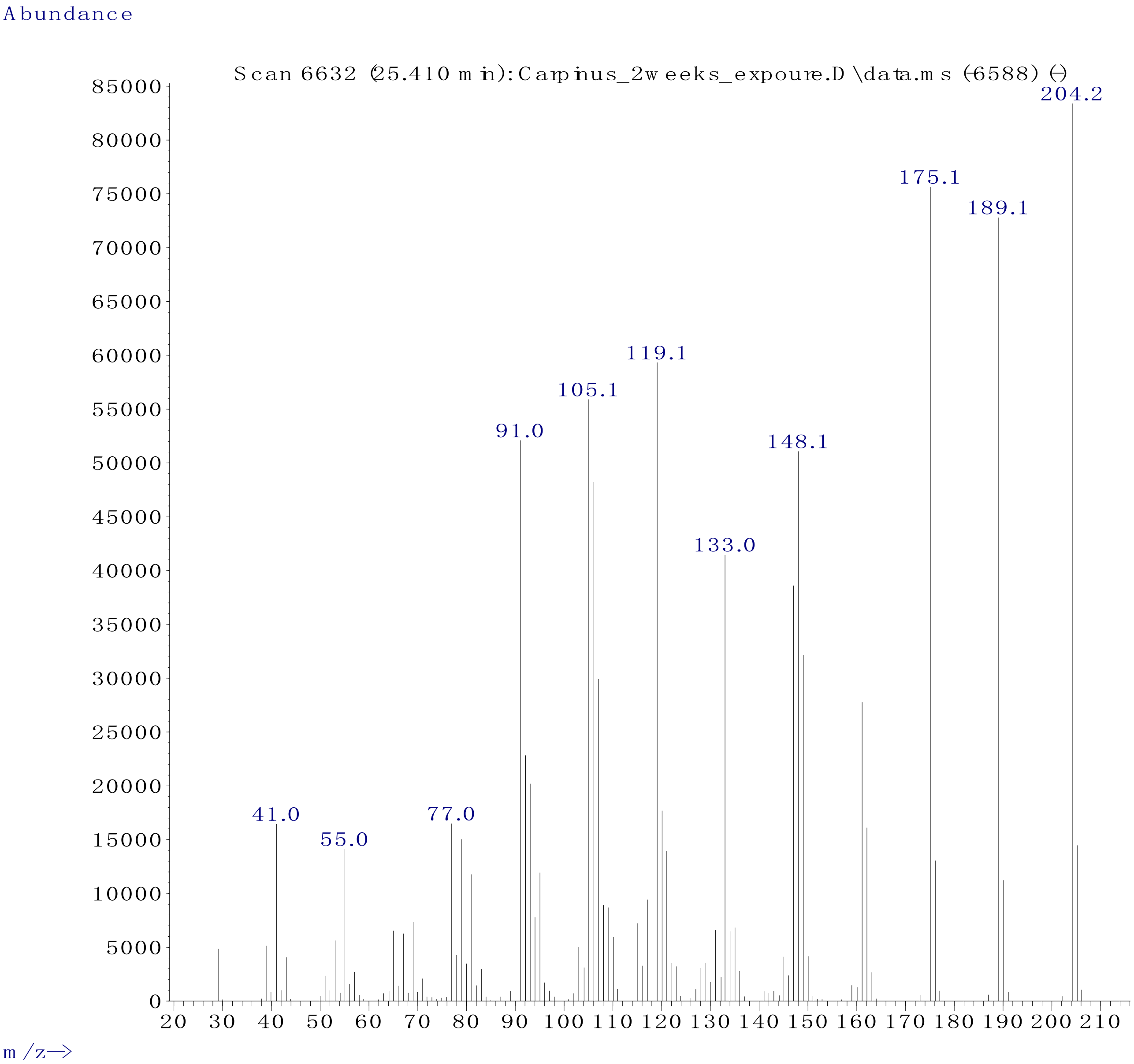

Unknown sesquiterpene-2, RI = 1307

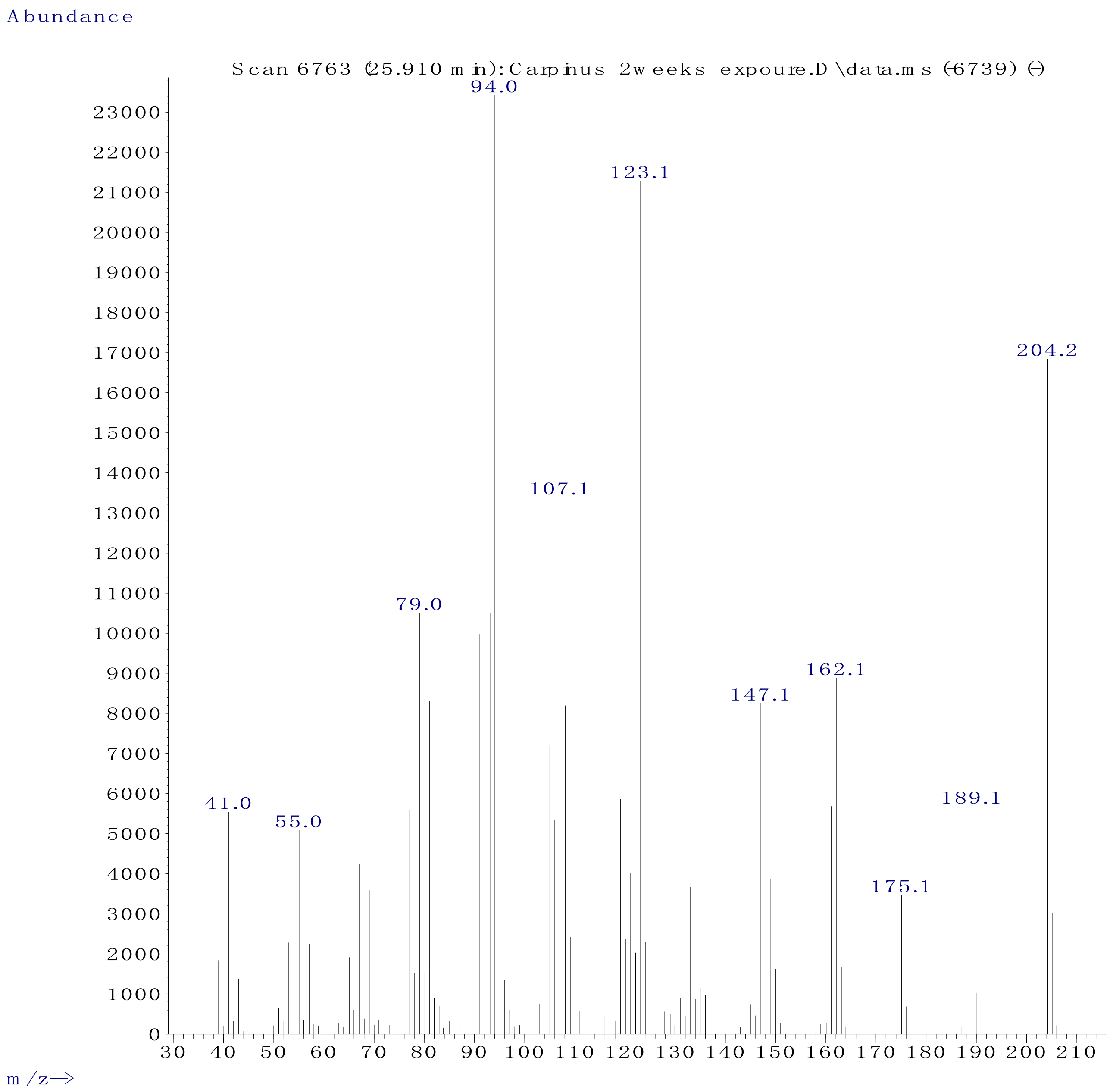

Unknown sesquiterpene-3, RI = 1318

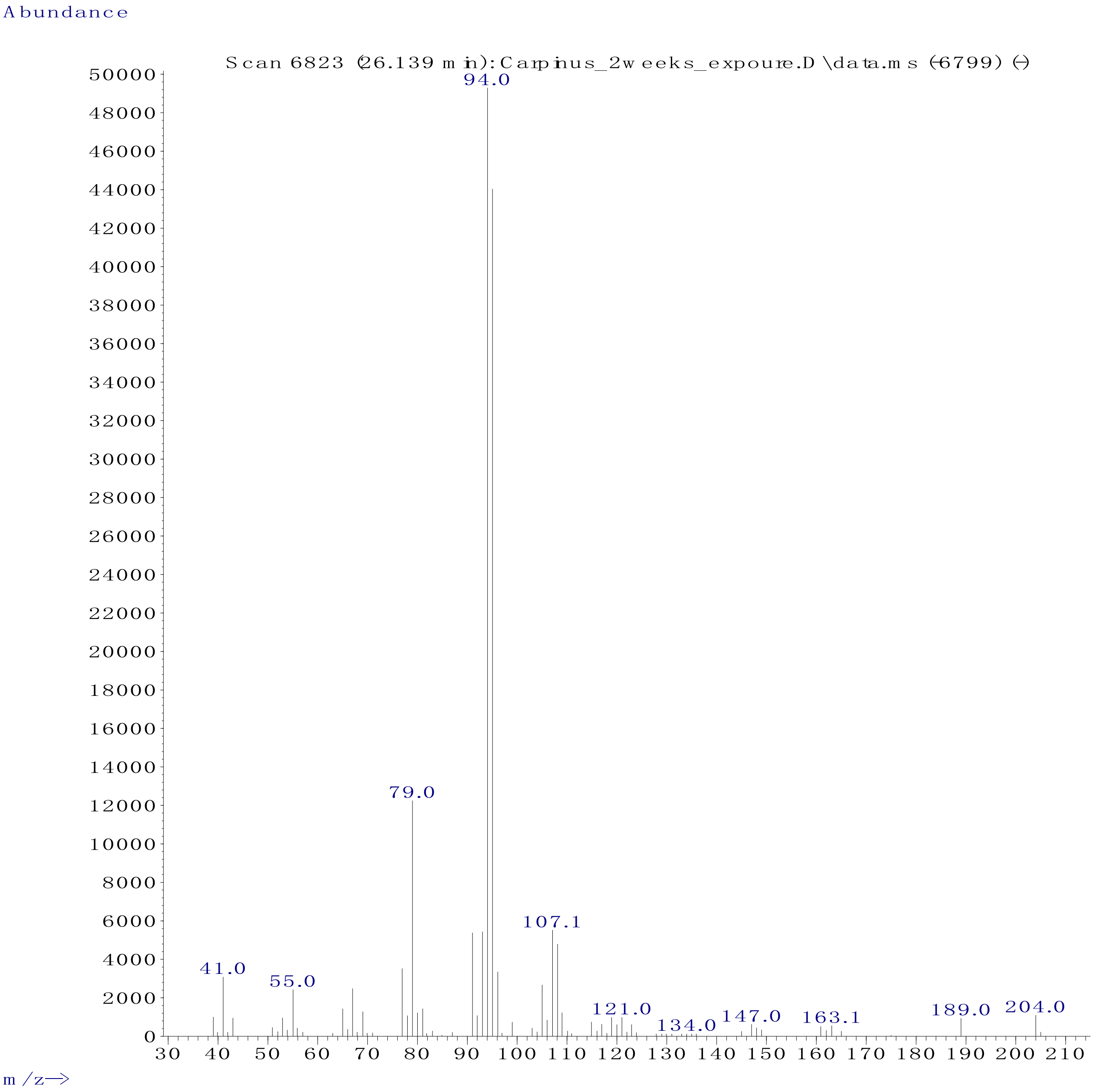

Unknown sesquiterpene-4, RI = 1324

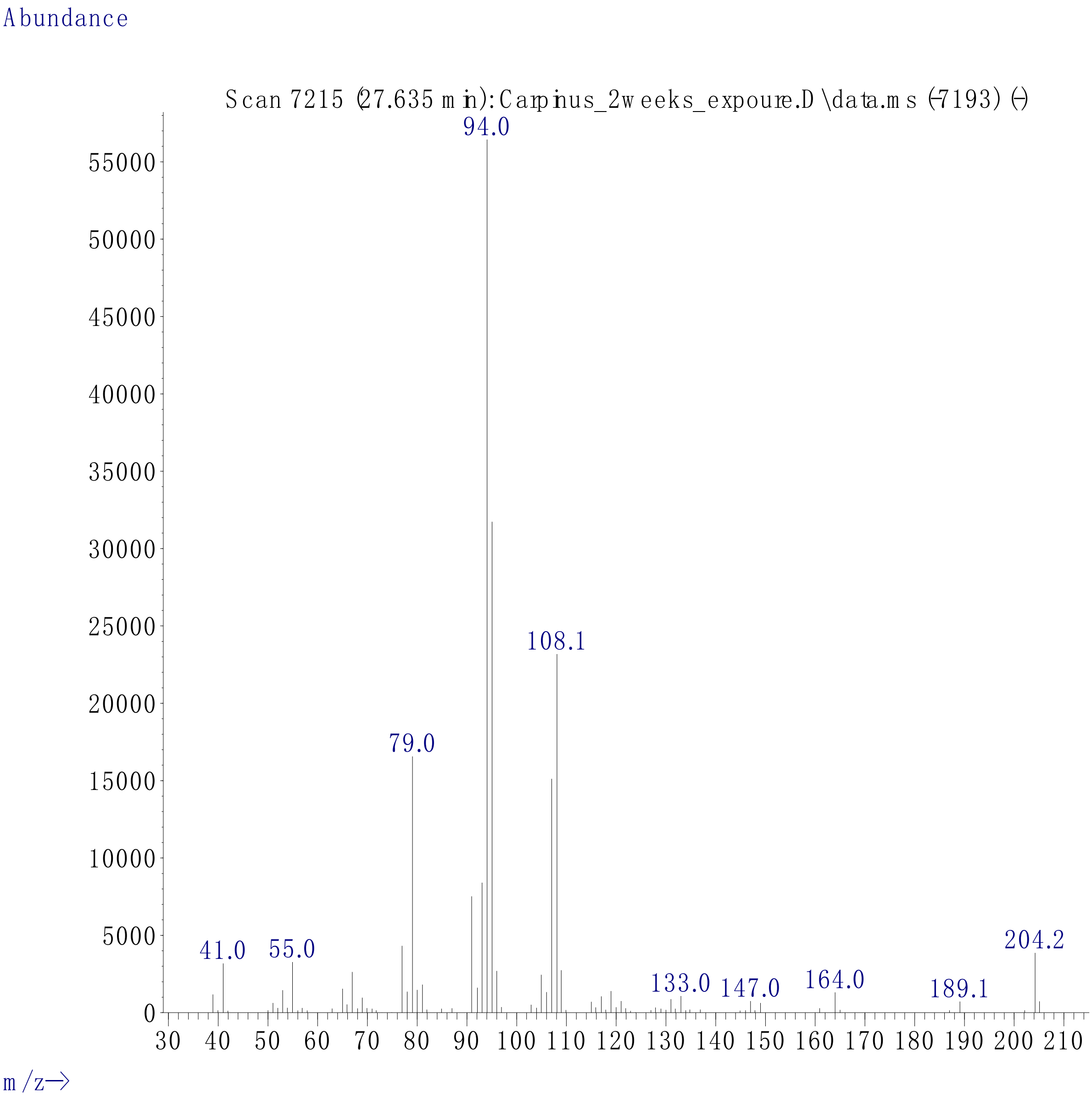

Unknown sesquiterpene-5, RI = 1345
